# A review of soybean processing byproducts and their use in swine and poultry diets

**DOI:** 10.1093/tas/txae063

**Published:** 2024-04-13

**Authors:** Katelyn N Gaffield, Robert D Goodband, Joel M DeRouchey, Mike D Tokach, Jason C Woodworth, Gordon Denny, Jordan T Gebhardt

**Affiliations:** Department of Animal Sciences and Industry, College of Agriculture, Kansas State University, Manhattan, KS 66506-0201, USA; Department of Animal Sciences and Industry, College of Agriculture, Kansas State University, Manhattan, KS 66506-0201, USA; Department of Animal Sciences and Industry, College of Agriculture, Kansas State University, Manhattan, KS 66506-0201, USA; Department of Animal Sciences and Industry, College of Agriculture, Kansas State University, Manhattan, KS 66506-0201, USA; Department of Animal Sciences and Industry, College of Agriculture, Kansas State University, Manhattan, KS 66506-0201, USA; Gordon Denny, LLC, Thornton, CO 80602, USA; Department of Diagnostic Medicine/Pathobiology, College of Veterinary Medicine, Kansas State University, Manhattan, KS 66506-0201, USA

**Keywords:** gums, poultry, soapstocks, soybean byproducts, soybean meal quality, swine

## Abstract

Due to its importance in animal feed, soybean meal has been extensively studied to optimize its use in livestock diets. Despite extensive research, the industry has not fully characterized specific areas of soybean processing such as the inclusion of soybean byproducts added back to soybean meal during processing. Soybean processing byproducts can encompass a large variety of materials including weeds and foreign material, soybean hulls, gums, soapstocks, lecithins, spent bleaching clays, and deodorizer distillates. Despite the potential for being added back to soybean meal when a crushing plant is integrated with an oil refinery, there is currently limited information on the composition of many of these soybean processing byproducts and their subsequent effects on soybean meal quality and animal performance. Therefore, there may be opportunities for a new area of research focused on soybean processing byproducts and their optimal use within the livestock feed industry. This review summarizes the current information on soybean byproducts with a focus on identifying the areas with the greatest potential for future research in swine and poultry nutrition.

## Teaser

Multiple byproducts are produced during soybean processing which may be included in monogastric diets through soybean meal or as an individual feedstuff. Soybean gums and soapstocks have the most potential value and need for additional research.

## INTRODUCTION

With high crude protein (44% to 48%) and energy (swine, net energy: 2,233 kcal/kg; poultry, apparent metabolizable energy: 2,473 kcal/kg), soybean meal serves as an affordable, high-quality protein source in animal feeds (Nahashon and Kilonzo-Nthenge, 2011; Lee et al., 2022; Stein, 2023). According to the United Soybean Board (2021), 78% of the total soybean meal produced was fed to poultry and swine (equivalent to 26.8 million metric tons). Emphasized by Ruiz et al. (2020), there are three areas of soybean meal research that continue to dominate the industry: amino acid digestibility, evaluation of antinutritional factors, and energy valuation. Despite extensive research, the industry has not fully characterized other specific areas of soybean processing such as the inclusion of soybean byproducts added back to soybean meal during processing.

Soybean processing byproducts can encompass a large variety of materials including weeds and foreign material, soybean hulls, gums, soapstocks, lecithins, spent bleaching clays, and deodorizer distillates (Table 1). Once produced, these byproducts may be disposed of as waste, sold to an identified market to add value, or added back to soybean meal. Despite the potential for being added back to soybean meal when a crushing plant is integrated with an oil refinery, there is currently limited information on the composition of many of these soybean processing byproducts and their subsequent effects on soybean meal quality and animal performance (Overland et al., 1993a, 1993b; Woerfel, 1995a, 1995b; Bruce et al., 2006). Therefore, there may be opportunities for a new area of research focused on soybean processing byproducts and their optimal use within the livestock feed industry. This need for understanding the optimal utilization of soybean byproducts will only increase as the need for soybean oil continues to grow to meet the demand for renewable fuels. This review aims to summarize the current information on soybean byproducts (weeds and foreign material, hulls, gum, soapstocks, lecithins, bleaching clays, and deodorizer distillates) with a focus on identifying the areas with the greatest potential for future research in swine and poultry nutrition.

**Table 1.** Summary of soybean processing byproducts and their use in monogastric diets

Soybean processing byproduct	Added back to soybean meal	Estimated amount added back to soybean meal	Common uses	Potential implementation in monogastric diets
Weeds and foreign material	No	—	Added to soybean hulls or disposed in landfill	None
Soybean hulls	No	—	Ruminant feedstuffs, wastewater treatment, and industrial applications	Dietary fiber source
Soybean gums	Yes	0% to 2%	Lecithin production or added back to soybean meal	Through soybean meal or as an energy source
Soybean soapstocks	Yes	0% to 2%	Acidulated for animal feed applications or added back to soybean meal	Through soybean meal, as an energy source, or as a pigment for poultry
Lecithins	No	—	Food applications, livestock feed, and pharmaceuticals	As an antioxidant or emulsification additive
Spent bleaching clays	Yes	Up to 0.50%	Added back to soybean meal as a flow agent, poultry feed ingredient, or disposal in landfill	Through soybean meal or poultry feed additive
Deodorizer distillates	No	—	Antioxidant or pharmaceutical applications	None (high demand in other markets)

## BACKGROUND ON SOYBEAN PROCESSING

The soybean oil milling industry developed out of the demand for soybean oil both for human consumption and industrial application (Shurtleff and Aoyagi, 2004). This demand is expected to grow as the production of sustainable biofuels is rapidly expanding (Wightman, 2021). This increase in demand will increase not only the production of refined soybean oil but also the byproducts produced throughout the oil extraction process. The main outputs of soybean processing are crude soybean oil and soybean meal. Crude oil is not viable for use in many applications, such as human consumption. Therefore, a sequence of further processing steps must be completed to refine the oil. Consequently, multiple additional byproducts are produced, which must find a market or appropriate disposal method.

Efficient soybean processing begins with the quality of incoming soybeans. Despite the importance soybean harvesting, handling, and storage have on the quality of the resulting products, soybean processors have minimal control over these initial factors (Erickson, 1995a). Understanding the variability of incoming soybeans allows processors to adjust processing steps appropriately. Even small changes in soybean oil or soybean meal production can have large impacts on soybean processing byproducts. For example, if the incoming soybean quality is lower, resulting in soybean meal close to the minimum protein target, there is very little opportunity for processors to add byproducts back to soybean meal. Adding byproducts devoid of protein to soybean meal would dilute the percentage of protein in that batch potentially leading to missed specifications and increased risk of claims. Therefore, this presents an immediate challenge when aiming to characterize the composition of byproducts and their use in animal diets as their presence in soybean meal is inconsistent throughout the industry and can be impacted by multiple factors.

**Figure 1.**
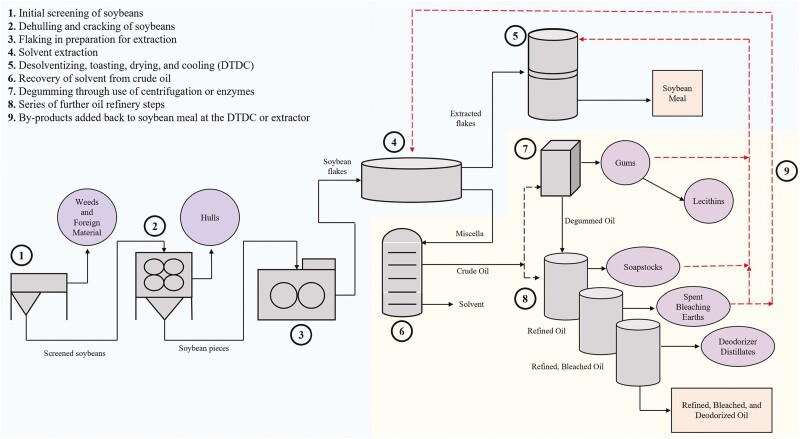
Soybean processing byproduct production throughout the soybean crushing and oil refining process.

Following arrival at the soybean processing facility, soybeans will go through a series of steps in preparation for oil extraction which may include cleaning, drying, cracking, dehulling, conditioning, and flaking (Woerfel, 1995b; Figure 1). Cleaning and dehulling soybeans are essential to maximize the protein content in soybean meal and increase extractor throughput for soybean processors (Woerfel, 1995b). Extractor throughput, which serves as a crucial economical factor for processors, is the rate at which a plant can run soybeans through the extractor in order to capture the oil. Once separated from the soybeans, hulls and other screenings such as weeds and foreign material become a byproduct stream for soybean processors to manage, further process, and market for other applications. Although weeds, foreign materials, or hulls are not commonly added back to soybean meal in modern production, the quantity of these residual products remaining in the soybean meal may vary. Therefore, cleaning and preparation of incoming soybeans using screens and aspiration have the potential to impact soybean meal quality. These screenings are frequently either added to the removed soybean hulls or disposed of as waste.

Following cleaning and dehulling, soybeans are then cracked into smaller particles and prepared for further processing. These small soybean pieces will then be flaked facilitating the rupture of the oil cells. Then the flakes are extracted using hexane (Lamsal and Johnson., 2007; Johnson, 2008). Due to the extraction efficiency and preparation of soybeans, it is now possible to produce soybean meal with as little as 0.8% residual oil (Johnson, 2008; NOPA, 2021). Once the oil is extracted, it will continue to be further processed resulting in refined soybean oil and several byproducts.

### Oil Processing

The liquid portion leaving the extractor is commonly termed miscella and is a combination of approximately 25% oil and 75% solvent. This mixture enters a series of evaporators and strippers to maximize the recovery of high-quality crude oil, while minimizing residual hexane (Demarco and Gibon, 2020). Depending on the desired product, crude oil can go through multiple refining steps including degumming, neutralization, bleaching, and deodorization (Erickson, 1995a).

Degumming is commonly the first step in oil refining and involves the removal of phosphatides from the oil. Depending on the specific plant and quality of incoming oil, degumming is not always implemented. There are multiple degumming processes that may be used including water, acid, dry, or enzymatic degumming (Dijkstra, 2010). Conventional degumming utilizes water to hydrate phosphatides that can then be centrifuged from the oil. Newer methods of degumming use enzymes, called phospholipases, to hydrolyze phospholipids (Yang et al., 2008). Although enzymatic degumming is thought to be a gentler method with a lower oil loss, conventional methods of degumming are still the most used. This process ultimately yields degummed oil and the byproduct known as soybean gums (Erickson, 1995b; NOPA, 2021).

The next step of oil refining is neutralization and can be implemented on either crude or degummed oil. Soybean processors often use neutralization through caustic refining to remove remaining phosphatides and neutralize free fatty acids (Erickson, 1995c). Caustic refining is defined as the treatment of an oil with an aqueous alkali solution, typically utilizing sodium hydroxide, also known as lye. Sodium hydroxide will react with free fatty acids in the oil creating soaps that can be removed through centrifugation coining the term “soapstocks” (Erickson, 1995c; NOPA, 2021). These soapstocks constitute an additional byproduct stream for soybean processors. As expected, a larger quantity of soapstocks will be produced if neutralizing crude oil that has not been through the degumming process, compared to an already partially refined, degummed oil. If plants utilize a physical refining step in lieu of caustic refining, free fatty acids will be removed through steam injection; therefore, not resulting in the generation of soapstocks (Gharby, 2022).

Following refining, oil can be bleached and deodorized to produce a final, fully refined oil product. Bleaching focuses on reducing oxidation products, removing remaining soaps and phosphatides, and extracting pigments. Acid-activated clays are commonly used during the bleaching process and can absorb pigments and other undesirable compounds (Erikson, 1995d; Abedi et al., 2015). Following bleaching, oils may be deodorized by the application of steam. Steam will volatilize any remaining undesirable compounds resulting in a colorless, odorless oil product (Zehnder, 1995). These final oil refining processes result in additional byproducts termed “spent bleaching clay” and “deodorizer distillate,” respectively (Erickson, 1995d).

Through these processing steps, multiple byproducts can be produced including weeds and foreign material, soybean hulls, gums, soapstocks, spent bleaching clay, and deodorizer distillates. Despite previous research and the long history of oil refining, there is limited literature outlining the composition and nutritional value of soybean processing byproducts. Therefore, there is a potential that the soybean processing and animal feed industries are overlooking an important component impacting soybean meal quality and the resulting animal feed products.

## SOYBEAN BYPRODUCTS IN NONRUMINANT DIETS

### Weeds and Foreign Material

Although representing a small portion of soybean meal, weeds and foreign material can have a notable impact on animal growth performance and health. Weeds and foreign material are composed of plant parts, broken beans, weed seeds, dirt, whole beans, pods, insects, and corn (Lin, 1996). Foreign materials can potentially impact the nutritional value of soybean meal, even at low inclusion levels, due to their high fiber content and potential for antinutritional factors. A study by Bruce et al. (2006) evaluated the impact of adding weeds and foreign material back at 2% of the soybean meal on nutrient digestibility in swine diets. They observed that both apparent ileal digestibility and standardized ileal digestibility of amino acids were lower when weeds and foreign material were added back to soybean meal. This could potentially be due to antinutritional factors including tannins and phenolics residing in the various weeds and seeds (Reed, 1995; Bruce et al., 2006). Additionally, having excess weeds and foreign materials in soybeans will dilute the nutrient contents as well as pose a potential risk to animal health through high levels of toxic contaminates (List et al., 1979). In modern soybean processing, it is uncommon to add weeds and foreign material back to the soybean meal. Weeds and foreign material screened from the soybeans are typically either added to pelletized soybean hulls or disposed of as waste.

### Soybean Hulls

Soybean hulls constitute approximately 5% of the total soybean and as a result, are the greatest amount of byproduct produced during soybean processing (Ferrer et al., 2016; Liu and Li, 2017). Soybean hulls are composed of varying amounts of cellulose, hemicellulose, lignin, pectins, and proteins. Therefore, hulls are lignocellulosic materials that are high in fiber and contain elevated peroxidases (Liu and Li, 2017). In addition to potential uses in industrial applications (treatment of wastewater and production of filaments), most hulls will end up in animal diets either through being added back to soybean meal or are in the form of loose or pelletized soybean hulls (Woerfel, 1995a).

Due to the low-energy and high-fiber content of soybean hulls, they are not commonly fed in swine diets (NRC, 2012). Past literature shows a consistent reduction in gain:feed ratio as nursery pigs are fed increasing levels of soybean hulls (Kornegay, 1978; Kornegay et al., 1995). This response is largely driven by the dilution of energy as more soybean hulls are added to the diet. Finishing pigs have an increased ability to ferment fiber compared to nursery pigs due to a maturation of the gastrointestinal tract and as a result are better equipped to handle additional fiber in the diet (Noblet and Van Milgen, 2004). Bowers et al. (2000) reported that finishing pig diets could include up to 3% added soybean hulls without affecting performance. Regardless of this increased ability to handle fiber, high levels of soybean hulls (between 6% and 30%) have resulted in reduced average daily gain (**ADG**) and feed efficiency in finishing pigs (Kornegay, 1978; Bowers et al., 2000; Stewart et al., 2013) unless maintaining the metabolizable energy per kilogram with the addition of fats or oils (Bowers et al., 2000). In addition to diluting the energy content of the diet, soybean hulls may also have a negative impact on digestibility. Dilger et al. (2004) observed that when feeding up to 9% soybean hulls, a 0.2% decrease in ileal digestibility of indispensable AA may occur for every 1% of added soybean hulls. This agrees with the data of Kornegay (1978) and Bruce et al. (2006) who observed decreases in energy and protein digestibility when soybean hulls were fed to growing pigs. Despite the diluted energy and potential reductions in digestibility, soybean hulls may have practicality in gestating sow diets, specifically within group housing systems. Fibrous ingredients, such as soybean hulls, have the potential to manage the satiety of gestating sows when fed at greater than 100 g of soluble fiber per day, ultimately helping limit aggression toward pen mates (Wensley et al., 2022).

Research evaluating soybean hulls in poultry diets has been inconsistent. Broilers fed high-fiber diets often result in a reduction in body weight gain (Jha and Mishra, 2021). However, there have been reported studies finding improvements in performance when soybean hulls are fed at a moderate level (approximately 5%) in poultry diets noting improvements in gain (Masoudi and Bojarpou, 2020; Tejeda and Kim, 2020). The variation in response to fiber in broiler diets is largely driven by the fiber source and inclusion level. In addition to performance, research has begun to focus on other benefits of feeding fiber sources, such as soybean hulls, in poultry diets. Like swine, one focus is on satiety. High-fiber diets have shown a potential to reduce cannibalism in poultry production systems, potentially serving as a feasible replacement for beak trimming in laying hens (Hartini et al., 2002). Furthermore, feeding soybean hulls has consistently shown improvements in organ development, indicated by increased gizzard and intestinal weights, potentially leading to the benefits in growth performance seen when fed at moderate levels to birds (Tejeda and Kim, 2020; Jha and Mishra, 2021). Additionally, other studies have reported beneficial aspects of soybean hulls in swine and poultry diets including stimulating growth of the gastrointestinal tract, reduced digesta passage rate, and improved intestinal health (Pascoal et al., 2015; Jha and Mishra, 2021). Therefore, current research should focus on the health-by-nutrition interaction associated with soybean hulls. Ultimately, soybean hulls are likely to be pelletized and marketed as a separate byproduct stream which can act as a dietary fiber source in monogastric diets but are most commonly used in ruminant feeds.

### Soybean Gums and Soapstocks

Soybeans generate the highest percentage of gums compared to other vegetable oils, making this an important byproduct for soy processors (Erickson, 1995b). The composition of gums is influenced by multiple factors including the quality of incoming soybeans, differences in processing procedures, and the age and efficiency of plant machinery. Although it will vary across the industry, the Practical Handbook of Soy Oil Processing and Utilization outlines the general composition of gums as 35% soybean oil, 17% phytogycolipids, 16% phosphatidyl choline, 14% phosphatidyl ethanolamine, 10% phosphatidyl inositol, and 7% carbohydrates (Erickson, 1995b). In addition to gums, soapstocks are another byproduct of the refining process. If left untreated, soapstocks will solidify and ferment leading to storage safety issues (Gerde et al., 2020). After the collection of soapstocks, they will either be neutralized or acidulated for animal feed applications. Once the product is acidulated, the composition is approximately 20% to 30% oil, 65% to 70% fatty acids, and 5% sterols and oxidized components (Erickson, 1995c).

Gums and soapstocks are both products commonly added back to soybean meal. It is most economical to include these byproducts back into the soybean meal to reduce the cost of transportation or disposal when a crushing facility is integrated with an oil refinery. Both gums and soapstocks will contain free fatty acids and phospholipids, ultimately allowing them to potentially be used as a source of energy in livestock feed (Gerde et al., 2020). One challenge with characterizing the effects of adding soybean gums and soapstocks back to soybean meal is the variation in composition and quantity. Current literature has not outlined the quantity of gums or soapstocks typically added back to soybean meal. This is likely because the quantities that are added back to the soybean meal at the desolventizing, toasting, drying, and cooling (DTDC) step are inconsistent and largely depend on the quality of the initial soybeans processed. Based on the ranges in analyzed nutrient compositions of soybean meal and personal communications throughout the soybean crushing facilities, an estimated inclusion of gums and soapstocks can range from 0% to 2%. The variation in the production of these two byproducts has been documented (Erickson, 1995b, 1995c; O’Brien, 2008) due to factors such as initial soybean quality, efficiency of the machinery, differences in the degumming process, and differences in the caustic refining process. However, there is currently no literature quantifying this variation across soybean processing plants.

Although there is currently limited data on composition, studies have aimed to evaluate the use of gums and soapstocks in monogastric diets. Soapstocks have been heavily studied by the poultry industry due to their potential as a low-cost energy source that could also successfully act as a pigment (Menge and Beal, 1973; Vieira et al., 2006; Borsatti et al., 2018). Specifically, poultry studies have indicated that replacing animal and vegetable fats and oils with dried soybean soapstock (Menge and Beal, 1973) or replacing degummed oil with acidulated soybean soapstock (Vieira et al., 2006) resulted in similar performance indicating soybean byproducts may act as an adequate energy source in poultry formulations. In addition to serving as a more affordable, but comparable source of energy, feeding soapstocks led to improved shank pigmentation indicating an additional benefit as a pigmenter (Menge and Beal, 1973). Broiler research has outlined a metabolizable energy value for acidulated soapstocks which ranges from approximately 6,700 to 7,500 kcal apparent metabolizable energy corrected for N/kg depending on the mixture of the product sourced and age of the bird (Gaiotto et al., 2000; Freitas et al., 2005; Borsatti et al., 2018).

The swine industry has also investigated the impact of soybean gums and soapstocks on growth performance and digestibility. Bruce et al. (2006) characterized the effects of soybean gums and soapstocks when added back during processing on the digestibility of soybean meal in swine. They aimed to utilize quantities of gums and soapstocks representative of those typically added back to soybean meal. This included evaluating both 1% and 3% added gums to soybean meal as well as 0.50% and 1.5% added soapstocks to soybean meal. They observed that increasing levels of gums and soapstocks added to soybean meal decreased the apparent ileal and standardized ileal digestibility of crude protein and amino acids (Arg, His, Leu, Lys, Phe, Val, Ala, and Gly). Additionally, they tested a combination of 3% gums, 1.5% soapstocks, and 2% weeds and foreign material added to soybean meal resulting in 6.5% total byproduct add-backs. This combination consistently produced the lowest apparent and standardized ileal amino acid digestibility when compared to individual byproduct treatments. Inversely, soybean byproducts did not appear to have an effect on apparent total tract digestibility of protein, fat, calcium, magnesium, or phosphorus when feeding soybean soapstock up to 10% of the diet (Atteh and Leeson, 1985) or apparent total tract digestibility of dry matter, crude protein, ether extract, and gross energy when feeding olive oil soapstock at 7.5% of the basal diet (Rojas-Cano et al., 2014). The potential decrease in digestibility of amino acids may be a result of chelating amino acids in the small intestine making them unavailable (Woerfel, 1995a). Additional research is needed to understand both the risks and benefits associated with added gums and soapstocks on nutrient digestibility in modern poultry and swine diets utilizing soybean meal reflective of today’s manufacturing processes.

Gums and soapstocks contain residual oil from the refining process and thus may serve as a potential energy source for swine and poultry. A study using up to 8% olive oil soapstock observed no significant effect on ADG or feed efficiency of growing pigs (47 kg). When olive oil soapstock was fed to finishing pigs (57 kg), both an increase in ADG and an improvement in feed efficiency were reported (Rojas-Cano et al., 2014). Lazor and Biocanin (1970) observed an improvement in ADG and feed efficiency when pigs were fed diets with 2% added sunflower soapstocks. The increase in performance seen in these studies was largely driven by the increased energy density of the diet. In contrast, Atteh and Leeson (1985) did not find significant differences in feed efficiency when feeding finishing pigs 5% soapstocks, but ADG decreased with the addition of 10% soapstocks. These diets were formulated to be isocaloric and contained similar levels of soybean meal as a protein source. Therefore, the cause of this decrease in performance is unknown and may indicate a limit for which soybean byproducts can be included in swine diets. Further research is needed to characterize the composition, variation, and nutritional value of soybean gums and soapstocks before they can be utilized in monogastric diets.

### Lecithin

Lecithin is derived from further processing soybean gums. If soybean processors are producing lecithins, the soybean gums produced during degumming will then be de-oiled and will go through alcohol fractionation (Gerde et al., 2020). The most difficult step in lecithin production is drying. The gums must go from approximately 50% to less than 1% moisture through a highly controlled heating process.

Lecithin can act as an emulsifier and antioxidant which has increased its demand in baking and food, cosmetic, and industrial applications (Erickson, 1995b). Lecithin may also be used in animal feeds. The addition of lecithins has the potential to interact with added fats which is thought to improve fat digestion. However, multiple studies have failed to find a positive response in both digestibility and growth performance when lecithins were added to swine diets (Overland et al., 1993a, 1993b; Soares and Lopez-Bote, 2002; Gatlin et al., 2005; Kerr and Shurson, 2017). A study feeding lysolecithin found improved digestibility of multiple fat sources; however, this response did not translate to improved growth performance (Jones et al., 1992). Although the emulsification benefits do not result in improved growth performance in swine, lecithins have shown benefits within the poultry industry. A review by Ullah et al. (2023) reported multiple benefits of lecithins in poultry diets including improved production, nutrient digestibility, and health status. Furthermore, they observed that as little as 2% added lecithin in poultry diets can lead to improved growth performance and lowered blood cholesterol levels (Ullah et al., 2023). Ultimately, the inclusion of soy lecithins in nonruminant diets will largely be driven by price with the benefits in poultry outweighing the benefits in swine diets.

### Spent Bleaching Clay and Deodorizer Distillate

Spent bleaching clay is the residual product of acid-activated earths used to remove oxidation products, remaining soaps, and residual pigments from soybean oil. The quantity of earths or clays produced during bleaching will change depending on oil quality (Gerde et al., 2020). Additionally, spent bleaching earths are difficult to handle and are extremely flammable (Erickson, 1995c). Although steps are taken to minimize oil loss, some residual oil remains in the used earths. Some oilseed processors will add spent clays to soybean meal as a flow agent up to the allowable inclusion of 0.5% (Smallwood, 2015; NOPA, 2021). Furthermore, processors can slurry the spent bleaching earths with crude oil and add it back to the extractor to maximize the recovery of oil (Dijkstra, 2020). These options are limited to oil refineries that are located next to a soybean crushing plant. If the earths are not added back to the soybean meal, oil refineries must find another method of disposal. Although there are technologies that allow for the regeneration of spent bleaching earths or the development of lick blocks for cattle, these are still relatively uncommon (Smallwood, 2015; Dijkstra, 2020).

Smith (1984) observed that bleaching clays from canola processing could be fed to swine at up to 10% of the diet for 17 d without affecting growth performance or feed intake. Another study fed up to 7.5% spent bleaching clay from canola refining to broilers without negative effects on health or growth and observed a positive effect on pellet hardness (Blair et al., 1986). Although past literature does not report any negative effects of spent bleaching clays in swine or poultry diets, this research has been limited with most studies conducted more than 30 yr ago. A review by Dijkstra (2020) summarized the best methods for handling spent bleaching earths. In this review, it was mentioned that adding bleaching earths back to soybean meal or disposal in a landfill are the most common methods. Adding bleaching earths back to soybean meal is not advised as it will increase the ash content of the soybean meal, consequently affecting its quality. However, for integrated soybean crush facilities and oil refineries, this is still the most economical option. Dijkstra (2020) advocates for the use of spent bleaching earths as a poultry feed ingredient when refineries are not adjacent to crush facilities. Further research is needed on how to best capture the energy value from this byproduct for use in nonruminant diets.

Deodorization is the final step in oil refining and removes volatile compounds using a distillate system to provide oil with a bland flavor, odor, and color. This process typically removes between 95% and 98% of tocopherols and sterols in the final oil (Woerfel, 1995a). The byproduct, deodorizer distillate, is valuable as it contains 4% tocopherol and 6% sterols (Gerde et al., 2020). Tocopherols are used as antioxidants and sterols are used to manufacture pharmaceuticals (Woerfel, 1995a). Due to their demand in human health and nutrition, these byproducts are more valuable in those industries and are less likely to be added to the soybean meal to be used as animal feed.

## CONCLUSION

Multiple byproducts are produced during soybean processing and oil refining which must find a market or proper disposal method. These products can include weeds and foreign material, soybean hulls, gums, soapstocks, lecithin, spent bleaching clay, and deodorizer distillates. Limited information exists throughout the soybean processing industry on the composition and quantities produced of each byproduct. While previous research has helped establish feeding values for some of these byproducts, more up-to-date information is needed. Both gums and soapstocks have shown a potential to act as an energy source in livestock diets, but there is limited information on the variation and quantities of gums and soapstocks produced and how they affect both poultry and swine performance. Therefore, these two soybean processing byproducts have been identified as having the most potential value and need for additional research.
